# A Smart Screening Device for Patients with Mental Health Problems in Primary Health Care: Development and Pilot Study

**DOI:** 10.2196/mental.9488

**Published:** 2018-05-28

**Authors:** Jan van Bebber, Rob R Meijer, Johana TW Wigman, Sjoerd Sytema, Lex Wunderink

**Affiliations:** ^1^ Interdisciplinary Center for Psychopathology and Emotion Regulation University Center for Psychopathology University Medical Center Groningen Groningen Netherlands; ^2^ Department of Psychometrics and Statistics Faculty of Behavioral and Social Sciences University of Groningen, Groningen Groningen Netherlands; ^3^ Rob Giel Research Center University Center for Psychopathology University Medical Center Groningen Groningen Netherlands; ^4^ Department of Education and Research Friesland Mental Health Services (GGZ Friesland) Leeuwarden Netherlands

**Keywords:** screening, primary health care, psychopathology, triage

## Abstract

**Background:**

Adequate recognition of mental health problems is a prerequisite for successful treatment. Although most people tend to consult their general practitioner (GP) when they first experience mental health problems, GPs are not very well equipped to screen for various forms of psychopathology to help them determine clients’ need for treatment.

**Objective:**

In this paper, the development and characteristics of *CATja*, a computerized adaptive test battery built to facilitate triage in primary care settings, are described, and first results of its implementation are reported.

**Methods:**

CATja was developed in close collaboration with GPs and mental health assistants (MHAs). During implementation, MHAs were requested to appraise clients’ rankings (N=91) on the domains to be tested and to indicate the treatment level they deemed most appropriate for clients before test administration. We compared the agreement between domain score appraisals and domain score computed by *CATja* and the agreement between initial (before test administration) treatment level advice and final treatment level advice.

**Results:**

Agreements (Cohen kappas) between MHAs’ appraisals of clients’ scores and clients’ scores computed by *CATja* were mostly between .40 and .50 (Cohen kappas=.10-.20), and the agreement between “initial” treatment levels and the final treatment level advised was .65 (Cohen kappa=.55).

**Conclusions:**

Using *CATja*, caregivers can efficiently generate summaries of their clients’ mental well-being on which decisions about treatment type and care level may be based. Further validation research is needed.

## Introduction

### Background

Mental well-being is fundamental to the functioning of communities and nations. However, the World Health Organization states that “[…] many people with mental health problems do not receive the treatment and care they need, despite the development of effective interventions” [1]. Matching the level of provided care to the client’s need for care is a difficult task because many factors have to be balanced simultaneously. Clients want access to the best care, but working hours of practitioners and clinicians are limited, and the interest of society is to keep care affordable. To reconcile these conflicting interests, various models of care have been proposed.

In the Netherlands, the structure of mental health care most closely resembles a stratified model, where “the initial treatment is selected based on the client’s treatment needs” [2]. The lowest level of mental health care is provided in general practices. Dutch general practitioners (GPs) are supported by mental health assistants (MHAs) who have a background in psychology, psychiatric care, or social work. MHAs are capable of treating light and or stable mental problems, and they can help to link clients to social care agencies for housing, employment, and or debt counseling. To get access to either generalist or specialist mental health care providers, clients need a referral from their GP. MHAs advise GPs on whether clients should be treated in general practices or whether they should be referred to either generalist or specialist mental health care providers. We use the term triage here to label the decision process just described.

### Aims of This Study

Psychological tests and questionnaires have long been used to provide valuable information to guide mental health care interventions. In this paper, a Web-based computerized adaptive test (CAT) battery (named *CATja*) is described that was specifically designed to screen clients in general practices for various forms of psychopathology, thereby facilitating triage. The construction of items banks [3-5] and the derivation of parameter estimates for these item banks have been described elsewhere [6-9]. In this study, we describe the development of *CATja* and report first results of a pilot study where MHAs implement the tool in daily practice.

## Methods

### Developmental Approach

Autonomy plays a crucial role in a person’s motivation, especially for those who are mainly intrinsically motivated [10]. Adopting *CATja* would require the MHAs to change their working routine in many ways, and because the best way to promote change is to provide those who are supposed to change with feelings of ownership of the new situation [11], we included MHAs in the developmental process. In addition, their expertise was highly valued. We organized regular meetings where we inventoried the opinions and ideas of MHAs and where we gave specific recommendations (such as testing adaptively to tap a broad range of constructs efficiently or how to safeguard clients’ privacy). Furthermore, these meetings enabled us to judge whether our plans would be supported. An important contribution by the MHAs was that we should not focus solely on deficiencies (eg, psychopathology), but should also pay attention to clients’ strengths (eg, positive psychological constructs). In addition, MHAs had a strong preference for blended care (ie, a combination of e-assessment and face-to-face interview). Besides the scores on various dimensions, each client is uniquely characterized by a specific combination of situational and environmental factors (eg, life events, motivation to change). Information on all these characteristics that make individuals unique was preferred to be obtained in face-to-face interviews. Furthermore, because a significant proportion of clients are treated by MHAs and the relationship between therapist and client is crucial for successful treatment [12], time spent on getting this auxiliary information during personal sessions is still spent in a valuable way, because these conversations probably strengthen the relationship between client and MHA.

### Computerized Adaptive Testing

In CAT, items that are presented to respondents are tailored to responses given to previous items. With each consecutive item, an updated person score is derived, and the item that increases measurement precision maximally for this score is used next. This process usually continues until a predefined measurement precision is reached. In CATs, much less items are needed to derive reliable scores compared with assessments with traditional questionnaires. For an introduction to CAT, see the study by Meijer and Nering [13].

### Content of the Alpha Version of CATja

The domains of psychopathology available in the alpha version were chosen based on (1) high prevalence in the target population (anxiety and depression), (2) the explicit wish of the envisioned end users (distress), and (3) severity of functional impairment (positive and negative symptoms of psychosis). Five psychopathology domains are currently available: anxiety and depression using the Patient Reported Outcome Measures Information System (PROMIS) item pools [9], positive and negative symptoms of psychosis based on the Prodromal Questionnaire [6], and the distress scale of the Four-Dimensional Symptom Questionnaire [7]. In addition, MHAs can assess the domains companionship and emotional support, using PROMIS item pools [8]. Thus, contrary to many existing eHealth screening tools [14], *CATja* incorporates domains of positive psychology as well as more severe symptoms of psychopathology (eg, hallucinations), and only uses items that are appropriate for a given client because of its adaptive testing routine.

### Sample Characteristics

We recruited MHAs by contacting Primary Care Consultants Northern Netherlands (ELAN), an organization that advises GPs in the north of the Netherlands on eHealth advancements. Four MHAs participated in the pilot study, and they assessed 31 MHAs’ clients in total (23 females). Clients were informed that their responses would be stored anonymously for research purposes, and they provided informed consent for this by selecting the hyperlink provided in the email that was sent to them by their MHAs. On average, clients were aged 30 years and 6 months (SD 12.2). All clients had achieved a high school degree, 3 graduated in applied sciences, and 3 graduated from university. With respect to relationship status, 9 clients chose the response option “living apart together,” 11 were living together, and another 11 clients reported to be single. Moreover, 12 clients reported to be still following education, 6 were looking for work, 4 were working part-time, and 9 were working full-time.

### Statistical Analyses

To get a first impression on how implementing *CATja* would change the information available to MHAs and how their decisions concerning clients’ triage would be affected, we did the following. For each domain on which clients were to be tested, we asked MHAs to estimate clients’quartile scores before administering *CATja*. These estimates were compared with the quartile scores computed by *CATja*. In addition, we requested MHAs to appraise expected treatment levels before testing their clients with *CATja* and to report final treatment levels advised after testing. We compared these initial and final treatment levels. The questionnaire used can be found in Multimedia Appendix 1. For all domains and treatment level advised, we computed coefficients of agreement.

## Results

The alpha version of *CATja* consisted of 3 interfaces (see [15]). When the domains and constructs to be assessed have been chosen, an invitation is sent to the client by email, which contains a hyperlink that leads to *CATja* ’s test administration interface. In this email, the client is informed that some information on their demographic background will be requested, and that their answers will be stored anonymously for research purposes. When responding, clients can change answers given to previous items, and revised scores are calculated. When finished, a report is automatically generated and sent to the MHA. In this report, several concepts that are essential for correct interpretation of the report are explained: the norm groups that served as reference for scores, the concept of quartiles (Q_i_), and the meaning of quartiles for psychopathology domains and positive psychological domains. For all psychopathology domains, low scores (Q_1_ and Q_2_) are indicative of healthy functioning, whereas for companionship and emotional support, high scores (Q_3_ and Q_4_) indicate healthy functioning. The main part of the report consists of a table with quartile scores for the domains administered. All items presented are given together with the response options chosen by the client at the end of the report.

Not all clients were tested on all domains; the number of subjects on which agreement could be based varied from 2 for negative symptoms of psychosis to 16 for anxiety and depression. In Table 1, the cross-tabulation of the quartile scores estimated by MHAs and the quartile scores computed by *CATja* is shown.

In 31 of 91 cases, clients’ scores estimated by MHAs before test administration and clients’ scores as computed by *CATja* were identical. The proportion of agreement equaled .35 (weighted kappa=.14). In case appraisals by MHAs and quartiles given by *CATja* were not congruent, MHAs’ appraisals were typically higher than quartiles computed by *CATja*. This trend was present only for the domains of psychopathology, not for the domains companionship and emotional support. Furthermore, agreement seemed to depend on the homogeneity of domain content. That is, agreement for the distress domain (2/15=.13) was lower than for anxiety (7/16=.44), depression (8/16=.50), and emotional support (6/14=.43). In 7 of 11 cases (weighted kappa=.57), the initial judgment of treatment level to be advised to clients and the final advice (after test administration) of treatment level were in agreement. In case of disagreement, initial treatment levels were always higher than final treatment levels advised to clients.

**Table 1:** Agreement between clients’ quartile scores appraised by mental health assistants before test administration and quartile scores computed by *CATja* (all domains and constructs).

Quartile estimated by MHA^a^		Quartile *CATja*			
		Q1	Q2	Q3	Q4
Q1		5	3	1	0
Q2		13	8	10	2
Q3		10	8	16	1
Q4		5	2	3	2

^a^MHA: mental health assistant.

## Discussion

The first results for the new screening device are promising, because the information obtained with it seems to add useful information to existing practice. Psychopathology domain scores as appraised by MHAs before test administration usually were higher than the domain scores reported by *CATja*. Furthermore, with respect to the treatment level advice, in case of no agreement, final treatment levels recommended to clients were always lower than the initial appraisals (before test administration). A tentative explanation for these findings would be that MHAs use the knowledge of the scores reported by *CATja* to lower the treatment levels they advise their clients. Under the assumption that the psychopathology domain scores computed by *CATja* are better estimates than the psychopathology domain scores appraised by MHAs, implementing *CATja* to determine the treatment level to be advised to clients would lead to less referrals to more specialized mental health care. Note that this preliminary finding, which would imply cost reduction, is opposite to what has been reported for other triage tools [16]. This result should be further tested in a study that includes many more clients in a randomized controlled treatment design where half of the participating MHAs use *CATja* and the other half does not. For all cases in which clients are referred to either generalist or specialist health care services, caregivers could be requested to rate the appropriateness of the referrals. On average, referrals for which *CATja* was used should be judged as more appropriate than those in the control condition. Another criterion for the incremental value of *CATja* would be to request clients to judge the degree to which they think their condition did improve since they contacted their GP.

## Appendix

Multimedia Appendix 1 Client form: Clients’ domain scores estimated by MHAs and MHAs’ appraisals of expected treatment levels.

## Abbreviations

CAT: computerized adaptive testing
GPs: general practitioners
MHAs: mental health assistants
PROMIS: Patient Reported Outcome Measures Information System
