# Supplementary material for: A Smart Screening Device for Patients with Mental Health Problems in Primary Health Care: Development and Pilot Study
Source: JMIR Ment Health. 2018 May 28;5(2):e41. doi: 10.2196/mental.9488 (PMC5996179; doi:10.2196/mental.9488)
Supplement: Multimedia Appendix 1 [file mental_v5i2e41_app1.pdf]

Appendix

A1. Form client

Name client/ client number:

The upper part of the form is not part of the data collected by the UMCG. Remove this part at the end of treatment, or at the end of the pilot

✂ -- ✂

**Test session requested**	
**Date:**	**__(dd) - __(mm)**
**Time:**	**__(hh) - __(mm)**

**Domain**	**Norm group**	**Expected result**[1]
**Positive symptoms of psychosis**	Clients generalistic MHC	Q1 Q2 Q3 Q4
**Negative symptoms of psychosis**	Clients generalistic MHC	Q1 Q2 Q3 Q4
**Anxiety**[2]	General population	Q1 Q2 Q3 Q4
	Clients specialistic MHC	Q1 Q2 Q3 Q4
**Depression**[2]	General population	Q1 Q2 Q3 Q4
	Clients specialistic MHC	Q1 Q2 Q3 Q4
**Friendship**	General population	Q1 Q2 Q3 Q4
**Emotional support**	General population	Q1 Q2 Q3 Q4
**Distress**	Clients generalistic MHC	Q1 Q2 Q3 Q4

	**Treatment in ...**		
**Expected level of care advised** [1]	General Practice	Generalistic MHC[3]	Specialistic MHC[3]
**Final level of care advised**	General Practice	Generalistic MHC[3]	Specialistic MHC[3]

In case expected and final level of care do not match, could you explain hereafter why not?

____

____

____

1 Mark the option that is appropriate according to your judgement.

2 The client´s scores on the domains anxiety and depression are compared with two norm groups: General population and clients specialistic mental health care.

3 Mental Health Care.
